# Human-Robot Interaction: Does Robotic Guidance Force Affect Gait-Related Brain Dynamics during Robot-Assisted Treadmill Walking?

**DOI:** 10.1371/journal.pone.0140626

**Published:** 2015-10-20

**Authors:** Kristel Knaepen, Andreas Mierau, Eva Swinnen, Helio Fernandez Tellez, Marc Michielsen, Eric Kerckhofs, Dirk Lefeber, Romain Meeusen

**Affiliations:** 1 Human Physiology Research Group, Vrije Universiteit Brussel, Brussels, Belgium; 2 Institute of Movement and Neurosciences, German Sport University, Cologne, Germany; 3 Center for Neurosciences, Vrije Universiteit Brussel, Brussels, Belgium; 4 Department of Rehabilitation Research, Vrije Universiteit Brussel, Brussels, Belgium; 5 Jessa Hospital, Rehabilitation Center Sint-Ursula, Herk-de-Stad, Belgium; 6 Robotics and Multibody Mechanics Research Group, Vrije Universiteit Brussel, Brussels, Belgium; 7 School of Public Health, Tropical Medicine and Rehabilitation Sciences, James Cook University, Queensland, Australia; Politehnica University of Bucharest, ROMANIA

## Abstract

In order to determine optimal training parameters for robot-assisted treadmill walking, it is essential to understand how a robotic device interacts with its wearer, and thus, how parameter settings of the device affect locomotor control. The aim of this study was to assess the effect of different levels of guidance force during robot-assisted treadmill walking on cortical activity. Eighteen healthy subjects walked at 2 km.h^-1^ on a treadmill with and without assistance of the Lokomat robotic gait orthosis. Event-related spectral perturbations and changes in power spectral density were investigated during unassisted treadmill walking as well as during robot-assisted treadmill walking at 30%, 60% and 100% guidance force (with 0% body weight support). Clustering of independent components revealed three clusters of activity in the sensorimotor cortex during treadmill walking and robot-assisted treadmill walking in healthy subjects. These clusters demonstrated gait-related spectral modulations in the mu, beta and low gamma bands over the sensorimotor cortex related to specific phases of the gait cycle. Moreover, mu and beta rhythms were suppressed in the right primary sensory cortex during treadmill walking compared to robot-assisted treadmill walking with 100% guidance force, indicating significantly larger involvement of the sensorimotor area during treadmill walking compared to robot-assisted treadmill walking. Only marginal differences in the spectral power of the mu, beta and low gamma bands could be identified between robot-assisted treadmill walking with different levels of guidance force. From these results it can be concluded that a high level of guidance force (i.e., 100% guidance force) and thus a less active participation during locomotion should be avoided during robot-assisted treadmill walking. This will optimize the involvement of the sensorimotor cortex which is known to be crucial for motor learning.

## Introduction

Robot-assisted gait training has a large potential to augment motor function and facilitate walking recovery in persons with neurological gait disorders [1–5]. It has some important advantages over manually assisted gait training such as the possibility to 1) increase the intensity and standardization of the training program, 2) offer a more complex multisensory stimulation of the human motor control system and 3) give extensive extrinsic feedback to the patient [6]. Yet, some reviews point out that there is little to no clear advantage of robot-assisted gait training over manually assisted gait training as far as rehabilitation outcome is concerned [7, 8]. One reason is that, until now, it is unclear how these active gait training devices should be designed, controlled and set (i.e., device settings such as the amount of assistance) to synergistically interact with the human body and optimally augment human performance [9, 10].

The assessment of how the human body interacts with robotic rehabilitation devices, which can be defined as human-robot interaction (HRI), has gained more and more attention over the past years and is a key aspect for the further optimization of the mechanical design and control of these devices [11]. HRI is often assessed through measures in the periphery such as kinematics, kinetics, muscle activity, metabolic energy expenditure, psychophysiological responses (e.g., heart rate, skin conductance & temperature, … etc.) or through the interaction torques with the robotic device [12–17]. Yet, bearing in mind that gait rehabilitation devices are primarily used to restore lost motor functions, assessing effects of HRI on the central level (i.e., central nervous system (CNS)) is important, as basic motor patterns are generated on the spinal as well as the supraspinal level [18, 19] and motor intentions and high-level adaptations of motor patterns originate at the supraspinal level [10, 20].

On the supraspinal level, cortical activation patterns have been monitored by means of electroencephalography (EEG) during lower limb movements [21, 22], actual walking on a treadmill (TW) [23–29] and robot-assisted treadmill walking (RATW) [30–35], see [36, 37] for a review. Most of these studies monitored event-related spectral perturbations (ERSPs) which reflect averaged dynamic changes in amplitude of the broad band EEG frequency spectrum as a function of time relative to a specific event, in the case of TW and RATW, the phases of the gait cycle [38]. The few studies which considered ERSPs during TW and RATW have mainly revealed power modulations in the alpha/mu (8–12 Hz), beta (12–30Hz) and low gamma (30–45 Hz) bands over the sensorimotor cortex related to the phases of the gait cycle, see [37] for a review and Table 1 for an overview. As compared to the studies presented in Table 1, where active RATW is mostly compared to passive RATW (i.e., with the same device settings) or standing, the purpose of this study was to look at possible effects of different amounts of guidance force of a robotic gait training orthosis (i.e., Lokomat) on brain activity and compare it to unassisted TW.

**Table 1:** Event-related spectral modulations in the cortex during TW and RATW.

Study	Mu 8–12 Hz	Beta 12–30 Hz	Low Gamma 30–45 Hz	High Gamma 45–100 Hz
*TW*				
Gwin et al. (2011)[23]	ERS during TSt	ERS during TSt	-	Broad intra-stride ERS/ERD changes
Severens et al. (2012)[25]	ERD during swing ERS just before IC and during DS	ERD during swing ERS just before IC and during DS	/	/
***Passive RATW*** ^a^				
Wagner et al. (2012)[30]	**-**	**-**	ERD during IC, TSt, PreSw, TSw	/
			ERS during MSt & MSw	
***Active RATW*** ^b^				
Wagner et al. (2012)[30]	**-**	**-**	ERD during IC, TSt, PreSw, TSw	/
			ERS during MSt & MSw	
Seeber et al. (2014)[31]	**-**	**-**	ERD during IC, TSt, PreSw, TSw	/
			ERS during MSt & MSw	
***TW vs standing***				
Severens et al. (2014)[39]	ERD	ERD	/	/
***Passive RATW vs standing***				
Nakanishi et al. (2014) [32]	ERS	-	/	/
Wagner et al. (2012)[30]	ERD	ERD	ERD	/
***Active RATW vs standing***				
Wagner et al. (2012) [30]	ERD	ERD	ERD	/
Seeber et al. (2014) [31]	ERD	ERD	-	/
***Active vs passive RATW***				
Wagner et al. (2012) [30]	ERD during swing, IC and TSt	ERD during TSt,, PreSw, ISw, MSw, TSw	ERD during PreSw	/
Solis et al. (2012) [34]	ERD	ERD	ERD	/

^a^ passive RATW = 100% guidance force, < 30% BWS, no participation

^b^ active RATW = 100% guidance force, < 30% BWS, active participation

/ = not included in the study; - = not significantly different; TW = treadmill walking; RATW = robot-assisted treadmill walking; BWS = body-weight support; ERS = event-related synchronization; ERD = event-related desynchronisation; IC = initial contact; MSt = midstance;TSt = terminal stance; PreSw = pre swing; ISw = initial swing; MSw = midswing; TSw = terminal swing; DS = double support

Six studies investigated ERSPs during RATW, all in healthy subjects. Nakanishi et al. (2014) compared passive walking in a gait rehabilitation device to standing and revealed an increase in power density of theta (4.0–7.9 Hz) and alpha bands. The authors suggested that this indicated a decreased arousal level and drowsiness during passive RATW [32]. Both Seeber et al. (2014) and Wagner et al. (2012) compared active walking in the robotic gait orthosis Lokomat (Hocoma AG, Switzerland) to standing [30, 31]. Active walking was defined as active participation during RATW with 100% assistance (i.e., 100% guidance force) and less than 30% body-weight support (BWS) [30, 31]. They found that, in the sensorimotor area, upper mu (10–12 Hz) and beta rhythms were suppressed (i.e., event-related desynchronization (ERD)) during the whole gait cycle compared to standing, while low gamma ERDs occurred at specific phases of the gait cycle, i.e., around the transition from stance to swing [30, 31]. Probably these mu and beta ERDs reflect the active state of the sensorimotor area during RATW compared to standing [30, 31]. Furthermore, Solis-Escalante et al. (2012) and Wagner et al. (2012) compared active to passive RATW (i.e., both at 100% assistance and <30% BWS) and revealed that the power spectral density (PSD) in the mu and beta bands in the foot/leg area of the sensory cortex was significantly reduced during active compared to passive RATW, indicating an increased activation of this area during active gait participation [30, 34]. Adaptation to a new gait pattern in a unilateral robotic orthosis was studied by Youssofzadeh et al. (2014) and indicated a strong involvement of the prefrontal area [35]. Finally, Wagner et al. (2014) also compared RATW to RATW in a virtual environment (i.e., both at 100% assistance and <30% BWS, but for the VE condition subjects had to execute force on the robotic gait orthosis to steer an avatar) and observed significant decreases in PSD of the 8–12, 15–20, and 23–40 Hz frequency ranges, as well as gait cycle related modulations in the frequency range 23–40 Hz [33]. These modulations were significantly reduced during specific phases of the gait cycle for RATW in a virtual environment compared to RATW, again indicating a more active state of sensorimotor areas [33].

In general, the functional meaning of these mu, beta and gamma modulations during RATW all point in the direction of maintaining an active movement related neuronal state during walking and processing of sensorimotor information [31]. None of these studies investigated the effects of variations in the level of assistance (i.e., guidance force) on event-related brain dynamics.

In the Lokomat, variations in the level of assistance a user receives can be achieved through adaptations in robotic guidance force (GF) [40]. GF is determined by the stiffness of the impedance controller which supplies a supportive force field within a haptic tunnel and smoothly corrects leg movements towards a specified trajectory. A value of 100% GF corresponds to a strict guiding (i.e., position control with stiff orthosis joints), while a value of 0% GF corresponds to free walking movements, meaning that full range leg movements are possible [16, 40, 41]. Thus, reducing GF allows for a more free and active participation to the gait cycle (i.e., the user can easily move away from the predefined gait trajectory) [16]. Until now, only the effects of GF on electromyography (EMG), kinematics, kinetics and metabolic energy expenditure have been investigated [16, 42, 43]. These few studies showed that the level of GF has no effect on oxygen consumption [42] and kinematics of the trunk and pelvis [43], but does affect muscle activity, kinematics and kinetics of the lower limbs: increased GF results in decreased muscle activity, increased joint range-of-motion (ROM) and increased joint range-of-forces [16]. These peripheral changes suggest that the effect of GF can possibly also be detected on the supraspinal level.

In summary, the goal of the present study was to reveal if changes in robotic GF affect gait-related brain dynamics. To this end we investigated ERSPs and PSD during RATW at 30%, 60% and 100% GF as well as during unassisted TW. It was hypothesized that, during RATW, there would be a significant decrease in the PSD of the mu, beta and gamma bands (ERDs) over the sensorimotor cortex with decreasing GF, indicating a larger involvement of the sensorimotor area when robotic assistance is reduced. Furthermore, it was expected that modulations in the beta and low gamma frequency bands related to specific phases of the gait cycle would be present.

## Materials and Methods

### Subjects

Eighteen young adult subjects with no neurological or locomotor deficits were recruited among the physical and occupational rehabilitation team of Rehabilitation Center Sint-Ursula, Jessa Hospital, Herk-de-Stad, Belgium. Eleven participants were included in this study (3 men, 9 women, mean age 28.2 SD 4.0 *years*, height 1.75 SD 0.09 *m*, weight 64.7 SD 7.7 *kg*), seven participants were excluded because of incomplete experimental trials or heavy artifact contamination of the EEG recordings.

### Ethics Statement

Participants gave their written informed consent to participate in this study. All experimental procedures were performed according to the standards set by the declaration of Helsinki for medical research involving human subjects and approved by the local medical ethics committee of the university, Vrije Universiteit Brussel, Belgium (BUN B1432008499) and Rehabilitation St.-Ursula, Herk-de-Stad, Belgium (12.11/fys12.02).

### Experimental Design

Participants completed four walking conditions of five minutes at a walking speed of 2 km.h^-1^. All subjects started with a baseline condition of five minutes of TW without the Lokomat gait orthosis. This was followed by three experimental conditions inside the Lokomat robotic gait orthosis with three different levels of GF, all under 0% BWS: 30%, 60% and 100% GF. The Lokomat is a robotic driven gait orthosis that gives assistance at the knee and hip joints in the sagittal plane and incorporates a motorized treadmill and BWS system [44, 45]. Individual adjustments of the Lokomat were made according to the common practice in clinical therapy with the help of physical therapists of the rehabilitation center having experience with the Lokomat. Safety procedures of the center required that all subjects also wore straps around the front foot to assist ankle dorsiflexion. The experimental conditions were randomized by stratified randomization based on increasing (30-60-100% GF) or decreasing levels of GF (100-60-30% GF). Participants were given instructions to follow the guidance of the orthosis and avoid pushing against it, to relax their arm muscles and rest them on the sidebars of the Lokomat, to avoid head movements and to look at a marker at eye level in front of them.

### Data Acquisition and Processing

Continuous EEG was recorded from 32 active electrodes (Fp1, Fp2, F7, F3, Fz, F4, F8, FC5, FC1, FC2, FC6, T7, C3, Cz, C4, T8, TP9, CP5, CP1, CP2, CP6, TP10, P7, P3, Pz, P4, P8, PO9, O1, Oz, O2, PO10) and sampled at 1000 Hz (BrainAmp DC & BrainVision Recorder, Brain Products GmbH, München, Germany). The electrical reference was located at FCz and the ground electrode at AFz, according to the international 10–20 system [46]. Electrode impedance was kept below 5 kOhm. A force sensing resistor placed on the subjects’ left and right heel detected left (L HS) and right (R HS) heel strike and was synchronized with the EEG data (BrainAmp ExG, Brain Products GmbH, München, Germany). EEG data were further processed offline using the EEGLAB v13.0.0b plug-in [47] for the Matlab 7.10 platform (MathWorks, Natick, MA, USA). The EEGlab ‘.study’ file with the original raw data can be found in S1 File. In a first step data were filtered (0.5 Hz high-pass, 100 Hz low-pass, 50 Hz notch filter), resampled to 512 Hz and segmented based on L HS. The segmented data were interpolated as such that the length of each gait cycle equaled 1200 ms. Next, epochs of 2000 ms were extracted (i.e., 1000 ms before and after L HS). Subsequently, artifactual epochs were removed using three steps. First, an automatic artifact detection procedure was applied, removing epochs with values a) ≥ ± 100 μV, b) ≥ 5 standard deviations of the mean kurtosis value, c) ≥ 5 standard deviations of the mean probability distribution, d) drifts ≥ 50 μV/epoch and R-square limit ≤ 0.3, and e) spectra deviating from the mean by ± 50 dB in the 0–2 Hz frequency window and by +50 or -100 dB in the 20–100 Hz frequency window. Second, the remaining epochs were visually inspected for artifacts. After this procedure, the average amount of epochs across all conditions and participants was 103 epochs with a standard deviation (SD) of 24.8 epochs. Third, an independent component analysis (ICA) decomposition algorithm (i.e., InfoMax) was performed on individual subjects over all conditions (i.e., multiple datasets were selected and concatenated in order to in order to give the same ICA weights to the different conditions) to parse EEG signals into spatially static, maximally independent components (IC) [48]. The DIPFIT function within EEGLAB [49] was used to compute an equivalent current dipole model that best explained the scalp topography of each IC using a boundary element head model based on the Montreal Neurological Institute (MNI) template (http://www.mni.mcgill.ca). ICs showing greater residual variance than 25%, or with a topography, time-course and power spectrum reflective of artifacts, were excluded from further analysis (202 ICs were excluded across all conditions and participants) [50–52].

In a next step, the remaining 144 ICs were clustered by use of principal component analysis (PCA) [53, 54]. Therefore, a preclustering array was implemented based on *k*-means clustering on vectors jointly coding differences between ICs in power spectra (dimension: 10, normalization weight: 1, frequency: 3–45 Hz), ERPs (dimension: 10, normalization weight: 1, latency: -1000 to 998 ms), dipole locations (dimension: 3, normalization weight: 10), ERSP (latency: -1000 to 998 ms, frequency: 3–45 Hz, dimension: 10, normalization weight: 1), and scalp topography (dimension: 10, normalization weight: 1). The resulting joint vector was reduced to 10 principal dimensions. This combined joint measure space over all subjects was clustered with a robust k-means algorithm into 11 clusters plus an additional cluster that contained outliers deviating by more than 3 standard deviations from the obtained cluster centers.

For each IC and each condition, the PSD and ERSP were calculated. Single-trial ERSPs were computed by averaging the difference between each single-trial log spectogram and the baseline (i.e., the mean IC log spectrum over all gait cycles per condition). Next, the PSD and ERSPs were averaged for each cluster and each condition [38]. To visualize event-related perturbations, significant differences from the baseline average gait cycle log spectrum were computed with a permutation method (p<.05) [47]. To assess differences between conditions, PSD and ERSPs were calculated in three frequency bands: mu (8–12 Hz), beta (12–30 Hz) and low gamma (30–45 Hz); using a common baseline (i.e., the average over all gait cycles) for the ERSP for each condition. A 4X1 one-way repeated measures ANOVA (REPANOVA) with factor “condition” (i.e., 1 = TW, 2 = RATW with 30% GF, 3 = RATW with 60% GF, 4 = RATW with 100% GF) was computed using a randomized permutation procedure in EEGLAB [55]. All reported p-values were adjusted for multiple comparisons using the EEGLAB implementation of false discovery rate (FDR) procedure [56]. Post-hoc tests were computed using paired permutation t-tests with correction for multiple comparisons (FDR). The significance level was a priori set at p<.05.

## Results

### ICA Clustering

**Fig 1:**
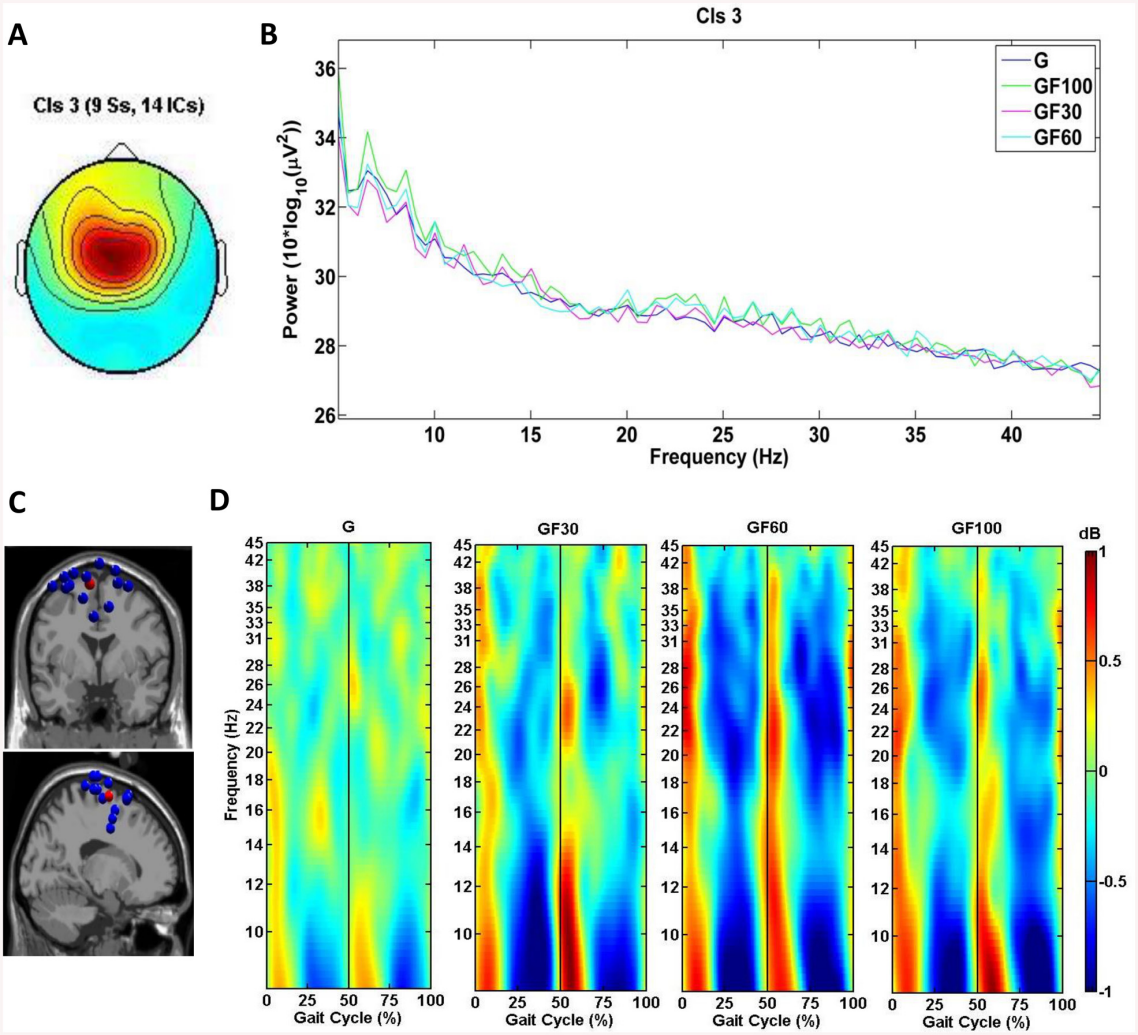
Scalp map, PSD, dipole locations and ERSPs for cluster 3, located in the midline PMC & SMA. (A) Cluster average scalp projection; (B) Gait cycle PSD for G, 30% GF, 60% GF and 100% GF; (C) Dipole locations of cluster ICs (blue spheres) and cluster centroids (red sphere) visualized in the MNI brain volume in coronal and sagittal views; (D) average cluster ERSP (3–45 Hz) plots showing significant changes in spectral power relative to the full gait cycle baseline (p<.05) for G, 30% GF, 60% GF and 100% GF. Non-significant differences relative to the full gait cycle baseline (p<.05) are masked in green (0 dB). The gait cycle starts and ends with R HS (i.e., 0 and 100%), the vertical line at 50% of the gait cycle marks L HS.

**Fig 2:**
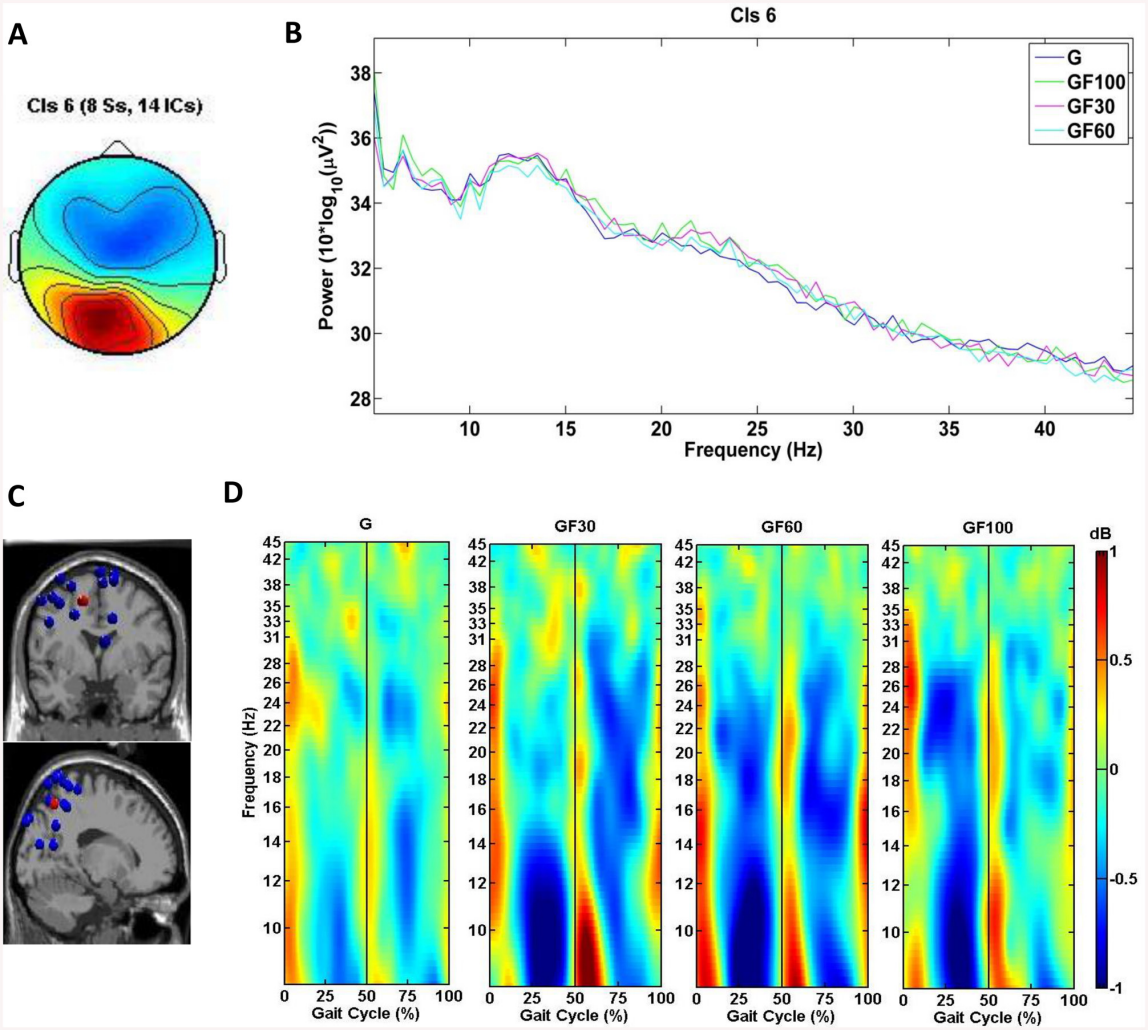
Scalp map, PSD, dipole locations and ERSPs for cluster 6, located in the left SA. (A) Cluster average scalp projection; (B) Gait cycle PSD for G, 30% GF, 60% GF and 100% GF; (C) Dipole locations of cluster ICs (blue spheres) and cluster centroids (red sphere) visualized in the MNI brain volume in coronal and sagittal views; (D) average cluster ERSP (3–45 Hz) plots showing significant changes in spectral power relative to the full gait cycle baseline (p<.05) for G, 30% GF, 60% GF and 100% GF. Non-significant differences relative to the full gait cycle baseline (p<.05) are masked in green (0 dB). The gait cycle starts and ends with R HS (i.e., 0 and 100%), the vertical line at 50% of the gait cycle marks L HS.

**Fig 3:**
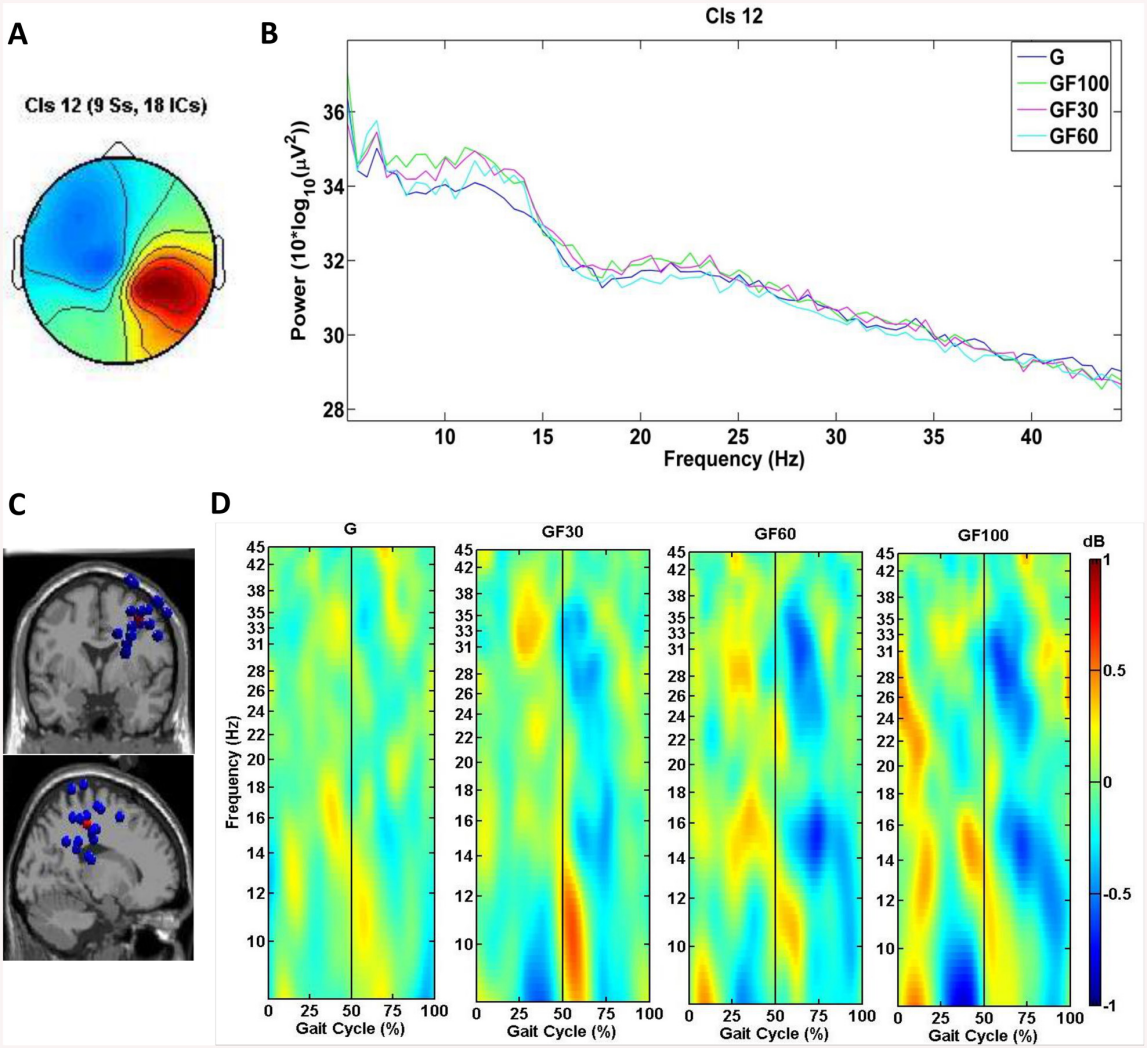
Scalp map, PSD, dipole locations and ERSPs for cluster 12, located in the right S1. (A) Cluster average scalp projection; (B) Gait cycle PSD for G, 30% GF, 60% GF and 100% GF; (C) Dipole locations of cluster ICs (blue spheres) and cluster centroids (red sphere) visualized in the MNI brain volume in coronal and sagittal views; (D) average cluster ERSP (3–45 Hz) plots showing significant changes in spectral power relative to the full gait cycle baseline (p<.05) for G, 30% GF, 60% GF and 100% GF. Non-significant differences relative to the full gait cycle baseline (p<.05) are masked in green (0 dB). The gait cycle starts and ends with R HS (i.e., 0 and 100%), the vertical line at 50% of the gait cycle marks L HS.

IC clustering analysis produced 11 active clusters and 1 cluster containing outliers, including 144 ICs. Table 2 shows the estimated dipole location of each cluster centroid in the MNI coordinate space, the corresponding Brodmann area (BA) and location of each cluster and the number of subjects and ICs included in each cluster. Some clusters were excluded from further analysis, based on the number of participants or ICs (i.e., < 8 subjects, < 11 ICs). From the remaining eight clusters, three target clusters were selected based on their region of interest for this study (i.e., the sensorimotor cortex). The target clusters were localized in the left premotor cortex (PMC) & supplementary motor cortex (SMA) (cluster 3, Fig 1C), the left somatosensory association cortex (SA)(cluster 6, Fig 2C) and the right primary somatosensory cortex (S1) (cluster 12, Fig 3C).

**Table 2:** Clusters of independent sources identified by ICA.

Cluster	# of ICs	# of Subjects	MNI Coordinates (x,y,z)	Brodmann Area (BA)	Location of Cluster Centroid
**Parentcluster 1**	144	11	/	/	/
**Cls 2 (outliers)**	4	3	/	/	/
**Cls 3**	14	9	-8,-2,63	L BA6	midline PMC & SMA
**Cls 4**	11	8	-51,33,16	L BA46	L PFC
**Cls 5**	9	6	-67,-41,-4	L BA21	L auditive cortex
**Cls 6**	14	8	-14,-58,50	L BA7	L SA
**Cls 7**	10	6	0,56,-8	/	/
**Cls 8**	13	9	64,-40,-4	R BA21	R auditive cortex
**Cls 9**	15	10	48,33,24	R BA9	R PFC
**Cls 10**	14	10	-35,-74,-28	/	/
**Cls 11**	10	6	-15,-17,8	L BA50	L thalamus
**Cls 12**	18	9	37,-25,45	R BA1	R S1
**Cls 13**	12	9	31,-84,-25	/	/

### Spectral Perturbations

Cluster 3, located in the midline PMC & SMA, revealed significant changes (p<.05) from baseline (i.e., the average over all gait cycles for each condition) relative to the phases of the gait cycle in the mu band during TW and in the mu, beta and low gamma band during RATW. For these frequency bands an ERD preceded the moment of L and R HS, while an event-related synchronization (ERS) occurred around L and R HS. This is displayed in the cluster average ERSP for each condition (Fig 1D). The one-way REPANOVA showed a significant effect (p<.05) in the ERSP at the mu band for G, 30% GF, 60% GF and 100% GF, yet post-hoc tests revealed no significant differences. This cluster also presented a significant effect (p<.05) in PSD for G, 30% GF, 60% GF and 100% GF in all three frequency bands (i.e., mu, beta and gamma) (Fig 1B), yet post-hoc tests showed again no significant differences.

Cluster 6 was located in the left SA and showed significant changes (p<.05) from baseline (i.e., the average over all gait cycles for each condition) relative to the phases of the gait cycle predominantly in the mu band for both TW and RATW. For RATW some significant spectral perturbations can also be seen in the beta band (Fig 2D). In general an ERD preceded the moment of L and R HS, while an ERS occurred around L and R HS (Fig 2D). The one-way REPANOVA showed a significant effect (p<.05) in the ERSP at the beta and low gamma bands between G, 30% GF, 60% GF and 100% GF, yet post-hoc tests revealed no significant differences. Cluster 6 also presented a significant effect (p<.05) in PSD for G, 30% GF, 60% GF and 100% GF in all three frequency bands (i.e., mu, beta and gamma) (Fig 2B). Post-hoc tests revealed significantly higher power over a small part (i.e., 9–10 Hz) of the mu frequency band for 30% GF compared to 60% GF (p<.05).

Cluster 12, located in the right S1, revealed less significant changes (p<.05) from baseline (i.e., the average over all gait cycles for each condition) relative to the phases of the gait cycle when compared to changes from baseline in cluster 3 and 6 (Fig 3D). Most of the changes occurred during RATW in the mu, beta and low gamma bands. The mu band followed the same pattern as previously described for cluster 3 and 6: an ERD preceded the moment of L and R HS, while an ERS occurred around L and R HS, however, as already mentioned, these perturbations are less pronounced compared to cluster 3 and 6. For the beta and gamma bands, no clear pattern related to the phases of the gait cycle can be observed (Fig 3D). No significant differences (p<.05) in the ERSP at the mu, beta and gamma band could be found between G, 30% GF, 60% GF and 100% GF. However, this cluster presented significant differences (p<.05) in PSD between G, 30% GF, 60% GF and 100% GF in all three frequency bands (Fig 3B). Post-hoc tests revealed a significant lower power in the mu frequency band for G and 60% GF compared to 100% GF (p<.05). Also, a significant lower power in the beta frequency band was observed for G compared to 100% GF (p<.05) (Fig 3B).

## Discussion

This study demonstrated gait-related spectral modulations in the mu, beta and low gamma bands over the sensorimotor cortex related to specific phases of the gait cycle. Furthermore, mu and beta rhythms were suppressed in the right primary sensory cortex during TW compared to RATW with 100% GF. Only marginal differences in the spectral power of the mu, beta and low gamma bands could be identified between RATW with different levels of GF.

### Clusters

Three clusters located in the sensorimotor cortex were identified: cluster 3 over the midline PMC & SMA as well as cluster 6 (i.e., L SA) and 12 (i.e., R S1) in the parietal cortex. The locations of these clusters are very similar to those found in the studies of Gwin et al. (2011)[23], Seeber et al. (2014) [31] and Wagner et al. (2012&2014) [30, 33]. More specifically, cluster 3 shows similarities in scalp maps, dipole locations and PSDs with clusters A in [30, 33]; cluster 6 corresponds well to cluster B in [33]; and cluster 12 shares characteristics with cluster D in [30] and cluster C in [33]. Previous research has suggested that activity in the PMC & SMA represents processes involved in motor planning [33, 57], while activity in the parietal cortex has been related to visuo-motor integration and bilateral coordination [33, 58].

The dispersion of the dipoles in the clusters identified in this study is somewhat larger compared to these in [30, 33], which may explain the slight differences in the locations of the cluster centroids. Nevertheless, the pattern of results with regard to active clusters over the sensorimotor cortex in this study is very similar to that obtained in other studies suggesting that it is possible to localize brain activity in specific sensorimotor areas related to walking with as little as 32 electrodes.

### ERSPs

Spectral perturbations in the mu (8–12 Hz), beta (12–30 Hz) and low gamma (30–45 Hz) bands from baseline (i.e., the average over all gait cycles for each condition) relative to the phases of the gait cycle were identified in all three clusters, although these were more prominent over the midline PMC & SMA (cluster 3) and L SA (cluster 6) and during RATW (Figs 1D–3D). ERSPs over the sensorimotor cortex related to the phases of the gait cycle have previously been identified during TW by Gwin et al. (2011) [23] and Severens et al. (2012) [25] and during RATW by Wagner et al. (2012&2014) [30, 33] and Seeber et al. (2014) [31]. In this study an ERD was generally observed preceding HS, when the leading leg was in the swing phase (i.e., following toe-off) and the trailing leg started pushing-off, while an ERS occurred around HS, during the double support phase. The ERSP in the cluster 6 corresponds well to the results of Gwin et al. (2011) [23] and Severens et al. (2012) [25], who reported the same pattern of mu and beta band modulations around HS and toe-off. It has been suggested that the modulations detected in these frequency bands are related to the control of sensory afferents [23]. In general an ERD in the mu and beta band corresponds to an activation of sensorimotor areas, while an ERS is typically associated with a deactivation [59, 60] or inhibition (i.e., mu ERS) [61] of the involved areas.

The ERD/ERS complex in cluster 3, the PMC & SMA, showed most of its activity in the mu band (8–12 Hz) and from 20–40 Hz (high beta, low gamma band). This is in line with Wagner et al. (2012&2014) [30, 33] and Seeber et al. (2014) [31] who reported ERSPs in the low gamma band (i.e., 24–40 Hz) during RATW, although the pattern of (de)synchronizations (i.e., ERD surrounding HS) is slightly different from this study (i.e., ERD preceding HS): the ERD in our study started around 25% and ended around 50% of the gait cycle while in Wagner et al. (2012) [30] the ERD started around 35% and ended around 60% of the gait cycle. These differences might be due to the additional effect of body weight support (<30% BWS) in the other studies [30, 31, 33]. Van Kammen et al. (2014) recently showed that adding BWS during RATW changes the muscle activity of the lower limbs [62]. Nevertheless, the portion of the ERD preceding HS is predominant in all studies and may represent processes involved to motor planning and coordination (i.e., PMC & SMA) during this phase of the gait cycle. Evidence for this has been put forward by Petersen et al. (2012) who observed significant coupling in the 24–40 Hz frequency range between the foot motor area and the activity of the tibialis anterior muscle during the swing phase, in preparation for HS of the leading leg [63]. However, the low gamma ERD could as well be related to the planning of the push-off phase of the trailing leg. Until today, corticomuscular coherence between the PMC & SMA and activity in the lower limb plantar flexors has not yet been studied. Thus, it remains to be determined which part of the gait cycle is linked to activity in the PMC & SMA during walking. It should be pointed out that Petersen et al. (2012) found this coherence over Cz, corresponding to M1, while in our study PMC & SMA were active. Yet, Feige et al. (2000) showed that multiple motor areas, including the PMC, are simultaneously involved in high-frequency corticomuscular coherence [64].

Although no significant differences could be determined between the ERSPs of TW and RATW and between RATW with different levels of GF, Figs 1D–3D display weaker gait-related modulations for TW compared to RATW conditions. Wagner et al. (2014) also found significantly reduced activity in gait-related modulations for cluster A (i.e., PMC & SMA) during RATW in a virtual environment where subjects had to execute additional force on the robotic gait orthosis to steer an avatar, compared to a normal RATW [33]. These results all indicate a higher level of involvement of the sensorimotor cortex during more active walking conditions. For cluster 3 in the PMC & SMA, this is particularly true for the 20–40 Hz frequency band (i.e., weaker gait-related modulations in this band during TW), confirming that a higher corticomuscular connection is present during TW compared to RATW with 100% GF [63]. This corresponds to the findings of Moreno et al. (2013), indicating that the activity of the tibialis anterior is significantly reduced during RATW compared to TW [16].

### Spectral Differences between TW and RATW

Power in the mu and beta band was significantly decreased in cluster 12 in the right parietal cortex during TW compared to RATW with 100% GF. This is in agreement with several other studies showing a significant decrease in PSD of the mu and beta band over the sensorimotor cortex during active compared to passive walking/pedaling, imagined walking or standing/rest [21, 22, 27, 28, 30, 31, 33, 34, 65]. The suppression of mu and beta oscillations is a general marker of an active movement-related neuronal state [31, 60, 66]. More specifically, these desynchronizations reflect increased cellular excitability in thalamo-cortical systems and can therefore be regarded upon as the electrophysiological correlate of cortical motor activity [60, 67, 68]. This is further confirmed by fMRI studies showing a more prominent increase in activation of the sensorimotor cortex during active compared to passive movements [69]. While the mu and beta band desynchronisation mostly occur simultaneously, there is recent evidence that these rhythms serve distinct functions [70]. For example, Petersen et al. (2012) found a corticomuscular coherence in the beta, but not in the mu rhythm [63] and in patients with Parkinson disease, Brown 2007 found that a pathological high beta band activity impedes the initiation and execution of movement [71]. Also, the topography of the mu ERD seems to be spatially less consistent (i.e., mostly spread out over the right parietal somatosensory area) than that of the beta ERD (i.e., more anterior, concentrated over the motor area) [31, 39, 60, 70, 72]. The desynchronisation of neurons firing in the mu band is possibly related to more general sensorimotor processes, while the beta ERD probably allows sensorimotor neurons to coordinate their actions in order to engage in a movement [70].

Activity in cluster 12 was asymmetrically concentrated over the right parietal hemisphere while it is generally thought that processing of somatosensory information is performed by brain regions contralateral to the side of the movement and thus should be symmetrically distributed during walking. However, there has been submerging more and more evidence that this is not entirely correct. Early observations from Mesulam (1981) and Nobre et al. (1997) showed that the left posterior parietal cortex processes information from the contralateral space, while the right posterior parietal cortex processes information from both spatial hemifields [73, 74]. Later on, Coghill et al. (2001) confirmed that the left inferior parietal cortex was only activated during right-sided stimulation, whereas the right inferior parietal cortex was activated during both left and right-sided stimulation [75]. A more recent and excellent review from Serrien et al. (2006) indicates that specialized functions for motor control are located in the right hemisphere. More specifically, the right hemisphere is crucial for closed-loop aspects of movement which are dependent on sensory feedback [76]. This corresponds very well to our results for cluster 12 which shows predominant activation in the right posterior parietal cortex, responsible for the processing of sensory information during walking.

In contrast to our hypothesis, there were almost no significant differences in PSD between the different levels of GF: 30% GF, 60% GF and 100% GF displayed similar PSD curves indicating that there is no difference in the involvement of the sensorimotor area when the level of robotic assistance changes. However, Moreno et al. (2013) found higher muscular activation for 20% and 40% GF in respect to 70% and 100% GF [16]. Yet, in their study 30% BWS was used, while in our study 0% BWS was implemented. Reducing body weight might have amplified the effects of GF (i.e., applying a force to an open kinetic chain will have more effect than to a closed kinetic chain).

Interestingly, there was also no significant difference in PSD between TW and RATW with 30% GF and 60% GF. This suggests that these RATW conditions might demand a similar involvement of the sensorimotor area as TW. Furthermore, Moreno et al. (2013) also showed that in neurologically intact subjects robotic-guided walking at various GF levels does not alter the basic locomotor control and timing of muscular activation patterns [16]. For future studies, it would be interesting to monitor the relationship between cortical activity and simultaneously recorded muscle activation and kinematics of the lower limbs during RATW.

### Methodological Considerations

Recently, Kline et al. (2015) were able to isolate gait-related movement artifacts during walking [52]. They found that EEG data recorded at slower speeds (i.e., 0.22–0.60 m.s^-1^) are less likely to be contaminated by movement artifacts. Moreover, the artifacts differ substantially across speeds, subjects and electrode locations which renders most of the existing artifact removal methods useless [52]. In order to actually subtract movement-related artifacts from brain activity during walking, they suggest to block the electrophysiological signals in some electrodes during data collection so that those electrodes record only the movement artifact [52]. We did not do this in our study and thus cannot be a 100% certain that all movement related artifacts are removed from the data. Yet, our subjects walked at a speed of 2 km.h^-1^ (i.e., 0.56 m.s^-1^) and thus our data are minimally contaminated by these type of artifacts according to the study of Kline et al. (2015). Furthermore, some of the movement artifact patterns were recurrent in the study of Kline et al. (2015), such as a desynchronisation in the 4–7 Hz range during double support (at slow speeds) [52]. As can be seen in Figs 1–3, this does not occur in our brain-related data.

Due to limitations of the Lokomat at higher speeds, the walking speed was set at 2 km.h^-1^. This could have implications for young healthy subjects who naturally walk at higher speeds. Studies have shown that, at slower speeds, the timing of muscle activation patterns changed and the amplitude decreased [77]. This could also have implications for brain activity: reduced muscle activity probably demands for less involvement of the sensorimotor cortex during walking, although this should still be confirmed. A good trade-off between avoiding movement-related artifacts and using speeds that are fast enough to be representative of typical human walking should be made [52].

When comparing RATW to TW it should be taken into account that there are some substantial differences between both which may alter the sensory feedback during walking. For instance, the gait orthosis imposes impedance to the limbs, which might constrain leg movements [62, 78]. Furthermore, movements in the Lokomat are restricted to the sagittal plane, thus reducing the natural degrees of freedom during walking. Nevertheless, according to Van Kammen et al. (2014) differences between TW and RATW can be kept to a minimum by imposing higher walking speeds (i.e., >1.8 km.h^-1^) and reducing the amount of body weight support [62]. In our study no body weight support was used and subjects walked at 2 km.h^-1^.

Furthermore, in this study we did not control for the level of active participation by means of EMG. It is possible that muscle activation of the lower limbs was similar between different levels of GF, although the EEG data suggest otherwise (i.e., at least for RATW with 100% GF compared to unassisted TW). Yet, the lack of significant differences for RATW at 30% GF and 60% GF might be because subjects didn’t adhere to level of assistance given by the orthosis and recruited similar levels of muscle activity during these conditions. Future studies, should try to control for the level of active participation by simultaneously recording EMG.

Although this study showed a very good correspondence to other studies [30, 31, 33], results might become more pronounced if more electrodes (i.e., >32) are used. This might influence the dispersion of the dipoles in the clusters and thus result in a more exact location of the cluster centroids. Nevertheless, Lau et al. (2012) were able to demonstrate that during walking as few as 35 channels may be sufficient to record the two most dominate electrocortical sources. This should be considered for practical reasons as multichannel arrays up to 256 channels take much longer to apply and might be more inconvenient for the patient/subject [79].

### Clinical Relevance and Implementations

In order to determine optimal training parameters for RATW, it is essential to understand how parameter settings affect locomotor control. This study showed that, in healthy subjects, power in the mu and beta bands was significantly reduced over the right primary sensory cortex during unassisted TW compared to RATW with 100% robotic assistance. This decrease might be related to the increased processing of sensorimotor information from the lower limbs. Yet, no significant differences in PSD between TW and lower levels of GF during RATW (i.e., 30% GF and 60% GF) were identified which might suggest that decreasing robotic-assistance provides training conditions that resemble unassisted walking. Furthermore, previous research has shown that motor learning, which is characterized by an increased mu ERD [31, 60], is further enhanced by increasing the task complexity, effort and sustained attention [60]. Therefore, low levels of guidance force (i.e., 30% GF, 60% GF) and thus active participation should be favored when possible during RATW although setting robotic assistance to zero may not be representative of common training conditions [62].

This study also emphasized that ERSPs and changes in PSD can be useful measures of active participation and motor performance during RATW and may help to establish functional improvements following interventions with RATW [30].

Some studies indicate that the effect of BWS might have a larger impact than the effect of GF during RATW [42, 43, 62]. In the current study, 0% BWS was implemented while different levels of GF were compared. Yet, no significant differences between different levels of guidance force were found. In a follow-up study, it would be interesting to investigate the effects of different levels of BWS on ERSP and PSD over the sensorimotor cortex.

The presence of ERSPs during walking might also be useful for the development of non-invasive brain-computer interfaces in order to control a robotic device. In their review, Castermans et al. (2014) have extensively discussed the approaches of how brain signals can be imbedded in the control loop of assistive devices [37]. Besides, Severens et al. (2014) recently demonstrated how the mu and beta ERD during actual and imagined walking can be successfully implemented in a BCI context to train a linear classifier in distinguishing walking from not walking [28].

## Conclusions

In summary, this study identified three active clusters located in the sensorimotor cortex during TW and RATW in healthy subjects. These clusters demonstrated gait-related modulations in the mu, beta and low gamma bands over the sensorimotor cortex related to specific phases of the gait cycle. Furthermore, mu and beta rhythms were suppressed in the right primary sensory cortex during TW compared to RATW with 100% GF indicating significantly larger involvement of the sensorimotor area during TW compared to RATW. In contrast to TW, almost no significant differences in the spectral power of the mu, beta and low gamma bands could be identified between RATW with different levels of GF. This finding may have important clinical implications with regard to parameter setting for RATW. It remains to be elucidated in future studies whether patients with neurological disorders exhibit similar effects of GF during RATW.

## Supporting Information

S1 File Original Raw Dataset in EEGlab ‘.study’ format. This file does not contain identifying participant information.
(STUDY)
